# Preservation by lyophilization of a human intestinal microbiota: influence of the cultivation pH on the drying outcome and re‐establishment ability

**DOI:** 10.1111/1751-7915.14007

**Published:** 2022-02-06

**Authors:** Regina Haindl, Lisa Totzauer, Ulrich Kulozik

**Affiliations:** ^1^ Chair of Food and Bioprocess Engineering TUM School of Life Sciences ZIEL‐Institute for Food and Health Technical University of Munich Weihenstephaner Berg 1 Freising‐Weihenstephan Germany

## Abstract

Faecal microbiota transplantation is an emerging medical concept for the treatment of gastrointestinal diseases. This concept, however, has disadvantages as low storability of stool and intensive donor screening. A solution to overcome these problems would be the preservation of an *in vitro* microbiota through freeze–drying. However, the influence of the entire preservation process, including cultivation and lyophilization, has not been assessed so far. In this study, the influences of the process steps cultivation, drying and re‐cultivation were determined with cell count, production of metabolites, microbial composition and diversity in the system as evaluation criteria. All pH conditions resulted in stable, culturable communities after re‐cultivation. Cell count, richness, diversity and microbial composition were affected by freeze–drying, but these effects were reversible and vanished during re‐cultivation. Hence, the re‐cultivated system did not differ from the system before drying. The metabolism, measured by short‐chain fatty acids as indicators, showed slight changes due to natural dynamics. Consequently, the cultivation prior to drying was identified to have more influence than the drying itself on the preservation process and therefore the biggest potential for optimization. Hence, the highest similarity with the initial stool sample was obtained with pH 6.0 ‐ 6.5 during cultivation.

## Graphical abstract

In this study, the preservation of an *in vitro* microbiota by lyophilization was conducted. Therefore, the influence of the cultivation pH value prior to drying and during re‐cultivation was investigated. Further, the ability to re‐establish a healthy stable system and the changes in cell count as well as metabolic and microbial behavior were of interest.

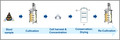

## Introduction

The human intestinal microbiota hosts a large and complex ecosystem with a high abundance of cells of up to 10^14^ CFU ml^−1^ and 400–1,000 different species (Eckburg *et al*., 2005; Gill *et al*., 2006). The microorganisms in the human gut belong to the phyla Firmicutes, Bacteroidetes, Actinobacteria, Proteobacteria and Verrucomicrobia. The two major phyla Firmicutes and Bacteroidetes represent 40–50% each of total bacteria. Actinobacteria (~ 2.5%), Proteobacteria (0.1–1%) and Verrucomicrobia (~ 0.1%) are less abundant and represent the minor phyla in the microbiota.

The complexity of this system is of great importance as alterations, compositional imbalances and a reduced diversity are often linked with several diseases (Holmes *et al*., 2011). One threatening disease linked with a decreased microbial diversity is the infection with *Clostridium difficile*, a spore‐forming microorganism that causes severe diarrhoea, nausea and fever (Abt *et al*., 2016). *Clostridium difficile* infections (CDI) often occur after extensive antibiotic treatment when the balance, richness and diversity in the gut microbiota are altered. Patients suffering from CDI have a disordered gut microbiota characterized by a decrease of *Bacteroides*, *Alistipes* and *Lachnospira* and an increase of opportunistic pathogens (Milani *et al*., 2016). Despite of the already disordered microbiota, the standard therapy is the treatment with antibiotics like metronidazole, vancomycin or clindamycin (Kelly and LaMont, 1998; Lagier *et al*., 2012). However, a threat of repetitive application of antibiotics is the occurrence of multiresistant strains and relapses of the disease. A total of 15–20% of patients with CDI relapse after the first antibiotic treatment and patients who suffered two or more previous relapses have a risk of 65% of further occurrences of severe CDI (McFarland, 2005; Sartelli *et al*., 2015).

Faecal microbiota transplantation (FMT) has been tested as an alternative treatment method to antibiotics, with recovery rates up to 90% (Gough *et al*., 2011; Aroniadis and Brandt, 2013). In this therapeutic concept, stool from a healthy donor is purified and transferred into the patient’s gut via colonoscopy, nasogastric tube or enema (Brandt and Aroniadis, 2013). The aim of the transfer is to re‐equilibrate the composition, diversity and thus to restore the function of a healthy microbiota in the patient’s gut. The restored microbial community inhibits *C*. *difficile* through competition for nutrients, suppression by antimicrobial peptides and bile‐acid‐mediated inhibition of spore germination and growth (Khoruts and Sadowsky, 2016). Further, the transferred metabolic products of the microbiota, short‐chain‐fatty acids (SCFAs), contribute by their anti‐inflammatory properties (Tedelind *et al*., 2007). In addition to the treatment of CDI, successful FMT applications including diseases like ulcerative colitis (Angelberger *et al*., 2013; Waller *et al*., 2021), Parkinson’s disease (Ananthaswamy, 2011; Xue *et al*., 2020) or multiple sclerosis (Borody *et al*., 2011; Li *et al*., 2020) have been reported. Nevertheless, the method has some limitations: ethical and safety concerns, a work and cost intensive donor screening prior to FMT and mainly the availability of suitable faecal material (Terveer *et al*., 2017; Zhang *et al*., 2018).

So far, different approaches to enhance the FMT concept were tested for the treatment of *C*. *difficile* infections (Chiu *et al*., 2021). The application of a processed, frozen and thawed donor stool resulted in recovery rates for CDI of up to 95% (Hamilton *et al*., 2012; Smirnova *et al*., 2019; Nicco *et al*., 2020). Further to that, different researchers investigated the treatment success with lyophilized faeces. A dried microbiota offered the additional advantages of less dependence on healthy stool donors, easy storability was well as high availability and flexibility for the administration in impaired patients. Vigvári *et al*., (2019) determined recovery rates for CDI of up to 83% through the application of lyophilized and resolved faeces via a nasogastric tube. When the powder was applied through capsules, the treatment succeeded for 84–100% of the patients suffering from CDI (Jiang *et al*., 2018; Staley *et al*., 2019). In our former study, a drying protocol for probiotics of the human intestinal microbiota was developed, where survival rates of up to 49% for the test strain *Bifidobacterium longum* were reached (Haindl *et al*., 2020). In this study, this drying protocol will be now used to preserve the entire microbiota. The drying outcome and ability to re‐grow and re‐establish can be tested by re‐cultivating the dried powder *in vitro*, which was the main purpose of this study. Next to the application of dried faeces, the rectal transfer of liquid *in vitro* infusion material has also already been investigated for the treatment of CDI (Garborg, 2015). Compared with the FMT concept, the application of an *in vitro* microbiota offers advantages like a technically easy to handle set‐up and operation, possibilities for variation and adaption of the system and further the lack of ethical considerations. In our previous studies, the establishment of an *in vitro* system, as well as the influence of the donor stool and the cultivation pH were investigated. An applicable system was established, creating a microbiota with slight differences in the distribution of phyla compared with the donor stool (Haindl *et al*., 2021a). Further, the influence of the donor itself was found to be small, with only some factors from the stool as, e.g., high abundances of single genera and metabolic characteristics sustain in the individual cultivated system (Haindl *et al*., 2021a). The choice of the cultivation pH had a major impact on the established *in vitro* system (Haindl *et al*., 2021b). The choice of a physiological cultivation pH during growth in a bioreactor system was found to depend on different criteria: a pH of 7.0 created a higher number of cells, whereas a lower pH of 6.0 resulted in a system with a phyla distribution more comparable with the donor stool and an higher abundance of health indicating microorganisms (Haindl *et al*., 2021b). The purpose in this study compared with previous studies was to determine whether the pH value may also have an influence not only during cultivation but also on the resistance of the *in vitro* cultured microbiota during freeze–drying and its ability to re‐establish. Bauer *et al*. (2012) showed that the fermentation conditions of single strains of probiotic cultures had an influence on the resistance against processing and thus on the survival after vacuum drying of the tested cultures. Next to freeze–drying as preservation method, Bircher *et al*. (2018) assessed the technical feasibility of the cryopreservation of an *in vitro* microbiota for FMT. Therefore, they cultivated purified faeces for 10 days in a continuous system at pH 5.7. After cell harvest and centrifugation, the cell pellet was frozen and stored at −80°C. After thawing, the microbiota was re‐cultivated for 24 h in a batch fermentation system. Consequently, the used preservation (freezing at −80°C) as well as re‐cultivation (batch) process and further the used media differed from this study. Bircher *et al*. (2018) detected a lowered metabolism in the re‐cultivated system with the concentration of all SCFAs being decreased. Further, when preserving without protectants, the abundance of butyrate producing microorganisms *Faecalibacterium* and *Roseburia* showed a strongly impaired growth.

The aim of this study was to build on these reports by applying the state of knowledge regarding preserved microbiota cultivated in bioreactors for FMT instead of isolation from stool donors. As the technical feasibility of the creation of an *in vitro* cultured microbiota was already demonstrated before (Haindl *et al*., 2021a), we aimed to demonstrate the technical feasibility of the preservation by lyophilization of an *in vitro* microbiome. This process might promote a high survival of health‐indicating microorganisms next to other advantages of a dried product as storability at ambient conditions and less weight. This study was conducted to investigate the whole processing chain including preparation of inoculum from stool, bioreactor cultivation, freeze–drying and testing survival and re‐cultivation of the dried culture in a bioreactor, which has not been investigated in this complete sequence by other researchers so far. Especially the ability of the dried product to re‐establish a stable system was of interest. The study investigates the influences of single process steps on survival and the ability of the technically produced cells to re‐grow and re‐establish a stable, cultivable community with a microbial composition comparable with the system prior to preservation as well as the initial faeces. The technical feasibility of maintaining high microbial abundance, diversity and richness was determined. Other studies mainly investigated the abundance of phyla or single genera after preservation (Bircher *et al*., 2018; Jiang *et al*., 2018). Here, the re‐establishment of several representative gut bacteria and health‐markers like *Clostridium* Cluster XIVa*, Bacteroides, Faecalibacterium*, *Bifidobacterium* and *Roseburia* after drying was determined. SCFAs as the metabolic products of a healthy microbiota were analysed because of their importance for the success and efficiency of treatment and crucial in restoring the metabolic balance of a disturbed intestinal microbiota (Tedelind *et al*., 2007). The purpose of the study was to determine the impact of the individual processing steps, i.e., cultivation with varied pH, freeze–drying and re‐cultivation of the dried microbiota, to possibly assess the most critical processing steps along the whole preservation process chain with a major influence on the outcome of the dried and re‐cultivated microbiota. By identification and adaption of these critical processing steps, a re‐established *in vitro* system with characteristics similar to the donor stool was intended to achieve with the perspective of applying this as an extension of the current FMT concept.

## Results and discussion

The approach was to assess the effects of single processing steps, i.e., cultivation, drying and re‐cultivation following rehydration and interactions between these steps. Therefore, samples were investigated throughout the whole process: after 120 h of cultivation (=before drying) and as well as during several time points in the re‐cultivation (=after drying) step.

### Effect of cultivation pH on drying outcome

At first, the influence and outcome of the drying process itself was identified by measuring residual moisture as well as survival of the cells by comparing the system after cultivation/centrifugation and the powder immediately after drying. It was aimed to create a stable product with a low moisture to ensure storability.

After cultivation, the broth was harvested, concentrated and the sediment frozen at −80°C. Afterwards, the drying process took place. After drying, residual moisture content, water activity and cell survival were determined (Table 1). For the residual moisture content, values between 5.0 ± 2.4% (pH 7.0) and 8.4 ± 0.7% (pH 6.5) were measured. Water activity was 0.14 ± 0.05 (pH 7.0), 0.15 ± 0.02 (pH 6.5) and 0.14 ± 0.02 (pH 6.0). All these results indicate a stable product. Microorganisms are more sensitive in an a_w_‐region of 0.3–0.5, where there is still too much water to bring them to a preserved state, but not enough water left to keep them in an agile state. Therefore, freeze‐dried microorganisms should always have a water activity below 0.3 to create a stable product (Higl *et al*., 2008). The data presented in Table 1 show that all tested cultivations resulted in lower a_w_‐values, i.e., all conditions applied led to stable and storable products.

**Table 1:** Residual moisture content, water activity and survival rate after cultivation at pH 7.0, 6.5 and 6.0.

	Cultivation pH value [–]		
	7.0	6.5	6.0
Residual moisture content [%]	5.0 ± 2.4	8.4 ± 0.7	7.6 ± 1.9
Water activity [–]	0.14 ± 0.05	0.15 ± 0.02	0.14 ± 0.02
Aerobic cell survival [%]	2.3 ± 0.6	5.5 ± 2.9	6.4 ± 3.3
Anaerobic cell survival [%]	0.1 ± 0.1	0.1 ± 0.1	0.9 ± 0.8

**Fig. 1:**
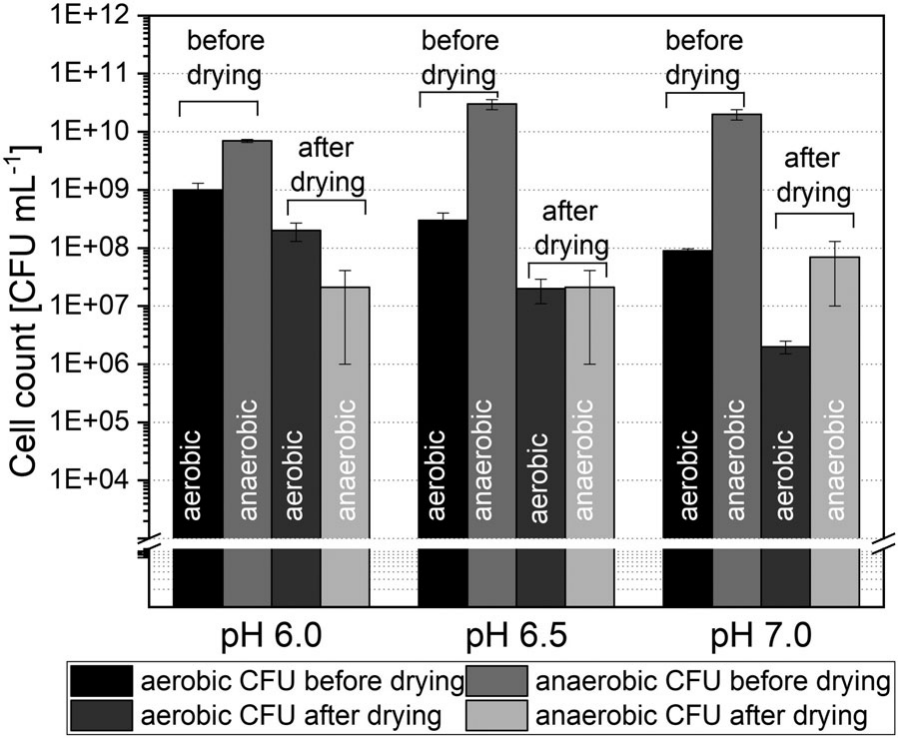
Comparison of aerobe and anaerobe cell count before and after drying for each cultivation pH value.

Figure 1 shows the aerobic and anaerobic cell count before (after 120 h of cultivation) and directly after drying for all investigated set‐ups. After cultivation and before the drying process, the aerobic cell count was between 9 · 10^7^ CFU ml^−1^ (pH 7.0) and 1 · 10^9^ CFU ml^−1^ (pH 6.0). After drying the cell count dropped steeply in the range of 10^6^–10^7^ CFU ml^−1^, resulting in aerobic survival rates between 2.3 ± 0.6% (pH 7.0) and 6.4 ± 3.3% (pH 6.0). For the anaerobic cell count, the cell count even decreased from numbers between 7 · 10^9^ CFU ml^−1^ (pH 6.0) and 3 · 10^10^ CFU ml^−1^ (pH 6.5) before drying down to 2 ± 2 10^7^ CFU ml^−1^ (pH 7.0 and pH 6.5) and 7 ± 6 10^7^ CFU ml^−1^ (pH 6.0) after drying.

The anaerobic survival rate therefore was only 0.9 ± 0.8% (pH 6.0) and 0.1 ± 0.1% (pH 6.5 and 7.0), indicating a significant decrease in anaerobic cell count. Compared with previous studies on the drying of single cell cultures, these survival rates are comparably low. For *Lactobacillus paracasei* and *B*. *longum*, which are also abundant in the human intestinal microbiota, higher survival rates of, e.g., 40% (Ambros *et al*., 2018) and 9% (Yang *et al*., 2012) after freeze–drying were reached. For these lower survival rates, several mechanisms may be in charge. The drying of a multicell suspension, where the drying protocol was not optimized for every microorganism in the complex mixture, may explain the significantly lower survival rates reached in this study. Further to that, single microorganisms in the microbiota are strictly anaerobic, whereas *Lactobacilli* and *Bifidobacteria* are more tolerant to short exposures to oxygen. Despite great efforts, the contact with oxygen could not be prevented entirely through the harvesting process, which may be another reason for the lower survival rates. Additionally, not all microorganisms of the human intestinal microbiota may be culturable after drying. It may be that these species survived the cultivation before drying, but due to their bad condition, could not survive the concentration, freezing and drying step and hence died after drying what consequently lowered the survival rate. Freeze–drying consists of the freezing and the drying step. Hence, some microorganisms may be sensitive to freezing and get even inactivated before the drying step itself. Bircher *et al*. (2018) have shown that *Roseburia* are not able to survive freezing and thawing. In this study, *Roseburia* were abundant in the cultivation step for all pH values but were below 0.01% abundance after re‐cultivation. Hence, a reason for the impaired growth may be the freezing step, but this needs further investigation. The water removal during freeze–drying is due to sublimation, which may also cause damages in the cells, especially in the cell membrane, that may lead to further inactivation. Consequently, several mechanisms like exposure to oxygen, freezing sensitivity and sensitivity because of water removal may be reasons that lower the survival rates here. As these mechanisms also may interact with each other, no clear statement can be made which mechanism leads to inactivation to what extent.

Further, the influence of the cultivation pH value on the drying outcome was tested by a one‐way ANOVA (*P* ≤ 0.05) followed by a Tukey *post hoc* analysis. It revealed no significant differences between the results for the residual moisture content as well as water activity and survival rate. Overall, it seems like the cultivation pH had no influence on the outcome directly after drying. In total, it has been shown that lyophilization has a severe impact on the survival directly after drying. Nevertheless, the influence on the ability to re‐grow and re‐establish a stable system must be considered and will be investigated in the following.

### Re‐establishment of an in vitro system after drying

After drying, a low survival rate of the cells was measured (Table 1). Nevertheless, these results only inform about the drying stress, but the survival rate directly after drying does not provide evidence for the ability of the cells to re‐establish and re‐grow during a re‐cultivation comparable with the initial bioreactor cultivation step before lyophilization. Further, the open question was how and what kind of microbiota was able to re‐establish. Therefore, the dried product was rehydrated and re‐cultivated for at least 70 h. For re‐cultivation, the same pH value as prior to drying was used. During re‐cultivation, cell count, metabolic production, microbial composition as well as diversity were measured for each experiment. In this section, the re‐establishment of the system cultivated and re‐cultivated at pH 6.5 are described in detail.

#### Cell count

**Fig. 2:**
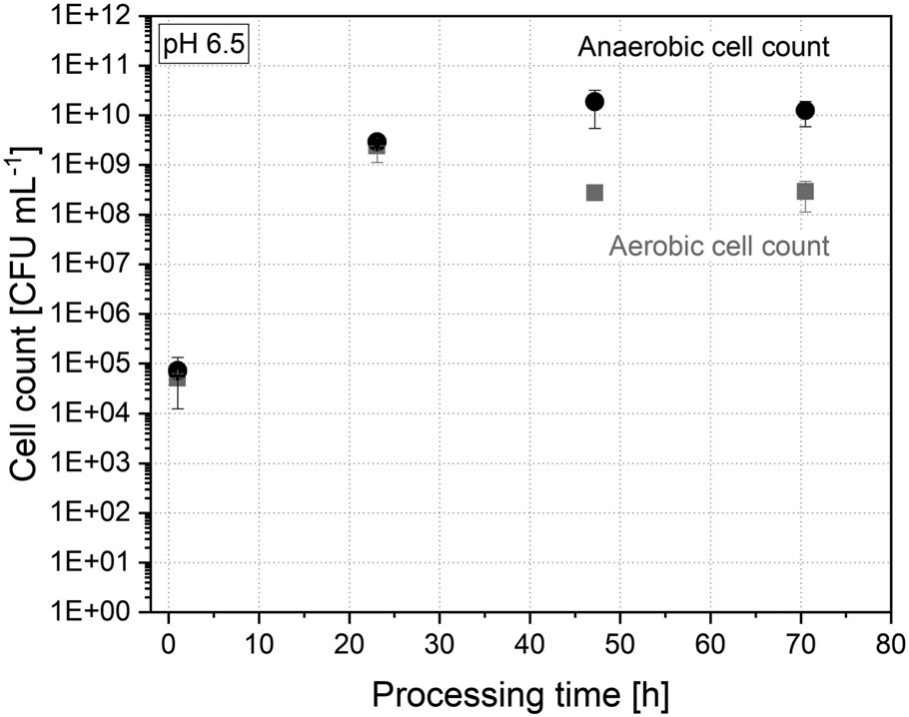
Evolution of the aerobic (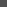) and anaerobic (●) cell count during the re‐cultivation after drying at pH 6.5.

The initial number of colony‐forming units prior to drying was 3 ± 0.3 10^8^ aerobic CFU ml^−1^ and 1 ± 0.7 10^10^ anaerobic CFU ml^−1^ (pH 6.5). Directly after drying, the cell count dropped and was determined with 2 ± 0.9 10^7^ aerobic CFU ml^−1^ and 2 ± 2 10^7^ anaerobic CFU ml^−1^. Due to the dilution by the cultivation media upon re‐inoculation, the cell count dropped further and reached 5 ± 1 10^4^ aerobic CFU ml^−1^ and 7 ± 6 10^4^ anaerobic CFU ml^−1^ after 1 h of processing time (Fig. 2). Nevertheless, the cell count was able to recover and regrow to a number of 3 ± 2 10^8^ aerobic CFU ml^−1^ and 1 ± 0.7 10^10^ anaerobic CFU ml^−1^ after 48 h and then remained in a stable state towards the end at 71 h. Compared with the microbiota prior to drying the cell count was able to recover completely and reached similar numbers (2 ± 1 10^8^ aerobic CFU ml^−1^ and 1 ± 0.3 10^10^ anaerobic CFU ml^−1^ in the stable system after 120 h of cultivation at pH 6.5 before concentration and drying).

Compared with other studies, when stool samples were preserved by freezing, the cell count irreversibly dropped about one log CFU ml^−1^ and could not reach the same level as before preservation (Carvalho *et al*., 2021). In this study, the aerobic as well as anaerobic cell count reached the same level as before the freeze–drying preservation step. This may be due to different cultivation conditions in the study of Carvalho *et al*. (2021, 2021) and this study that resulted in lower cell counts. Further, during freezing, intracellular ice can be formed and the increase in solute concentration imposes osmotic stress to the cell (Santivarangkna *et al*., 2008). This mechanisms resulted in irreversible damages in the followed thawing process while they seemed not to occur during freeze–drying and sublimation.

#### Metabolic production

To investigate the ability of the dried microbiota to re‐establish with a working metabolism, the powder was rehydrated and re‐cultivated in the *in vitro* system again. The metabolic production was measured by the concentration of overall SCFAs in the broth as well as the main metabolites acetate, propionate, butyrate and iso‐valerate.

**Fig. 3:**
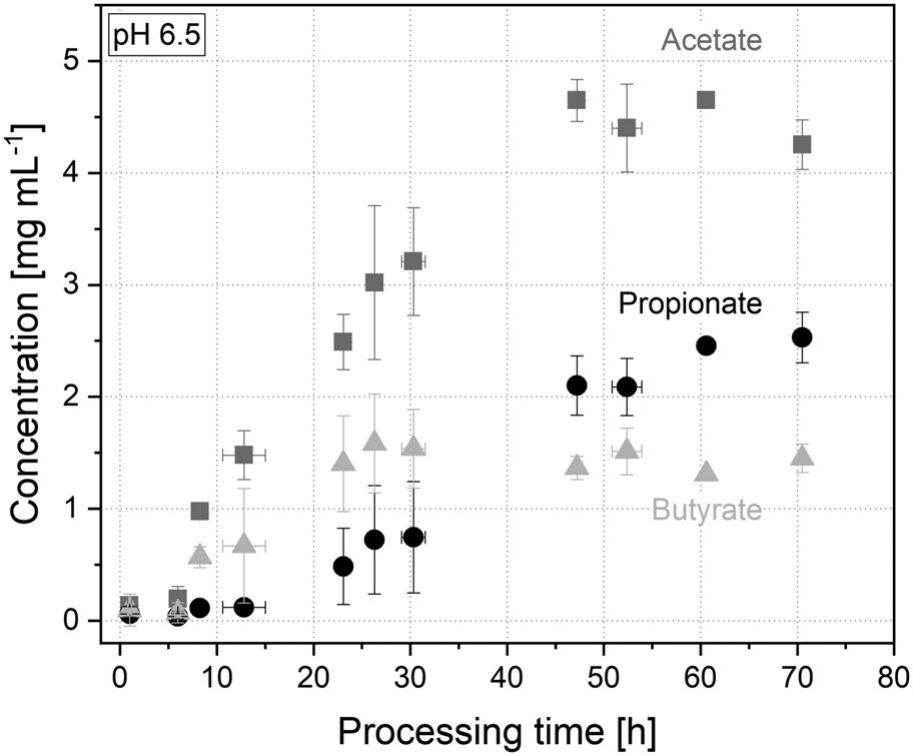
Concentration of acetate (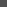), propionate (●) and butyrate (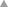) during re‐cultivation at a cultivation pH of 6.5.

In total, the sum of SCFAs started to increase after 6 h when the cells started to grow and metabolize. They increased continuously until a plateau and stable state was formed after 47 h at a total concentration of all SCFAs at 8.25 ± 0.60 mg ml^−1^. After re‐inoculation, the concentration of acetate in the broth was constant for the first 6 h at 0.20 ± 0.11 mg ml^−1^. As shown in Fig. 3, the concentration then steadily increased to 4.65 ± 0.19 mg ml^−1^ (47 h) and finally plateaued at a level of approximately 4.5 mg ml^−1^.

**Fig. 4:**
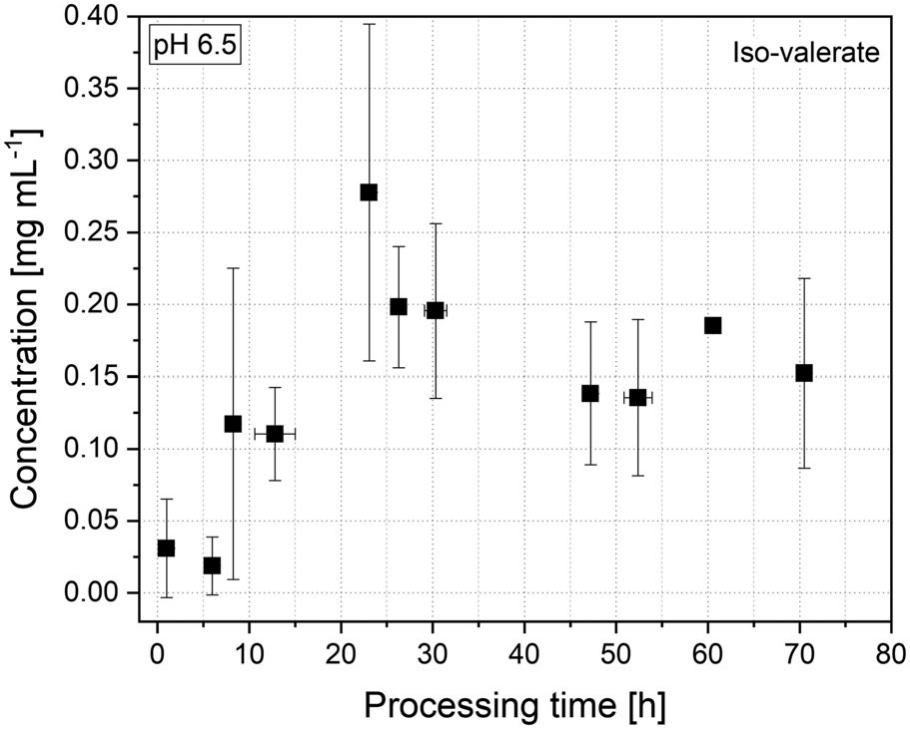
Concentration of iso‐valerate during re‐cultivation at a cultivation pH of 6.5.

The concentration of propionate was low in the beginning (0.06 ± 0.07 mg ml^−1^, 1 h) and started to increase after 23 h (0.49 ± 0.34 mg ml^−1^) until a stable state was reached after 61 h at a concentration of 2.46 ± 0.02 mg ml^−1^. The production of butyrate started earlier compared with propionate, after 6 h with a concentration of 0.07 ± 0.09 mg ml^−1^, increased and plateaued after 26 h at a concentration of 1.58 ± 0.44 mg ml^−1^. The concentration of iso‐valerate in the broth behaved similarly to the production of butyrate (Fig. 4). It stayed constant at a low level for the first 6 h (0.02 ± 0.02 mg ml^−1^) and then started to increase until a stable state was reached after 47 h at a concentration of 0.14 ± 0.05 mg ml^−1^. In total, the re‐cultivated microbiota showed a working metabolism after 6 h. First, acetate, butyrate and iso‐valerate were produced, whereas the production of propionate started later after 23 h of re‐cultivation. This gives the hint that a variety of different microorganisms started to grow at different points of re‐cultivation metabolizing different acids.

All values in the metabolic production plateaued after a certain time, indicating that a stable system was formed under steady state conditions. Along with the total amount of SCFAs, the ratio between acetate, propionate and butyrate is a marker for human health. Ratios from 3:1:1 (acetate: propionate: butyrate) to 10:2:1 are considered as typically healthy (Macfarlane *et al*., 1992; Rowland *et al*., 2018). Here, in the stable system (> 70 h), a ratio of 4:3:1 was reached indicating a healthy contribution. Other researchers reached ratios, depending on the cryoprotectant, in a similar range of 10:2:8, 10:4:13 and 10:5:4 (Bircher *et al*., 2018). Consequently, the concentration of SCFAs was in a comparable range with other studies and indicates that the metabolic production and behaviour could be restored after the drying process. Overall, the *in vitro* system after drying still represents a functional microbiome with a regularly working metabolism.

#### Microbial composition

**Fig. 5:**
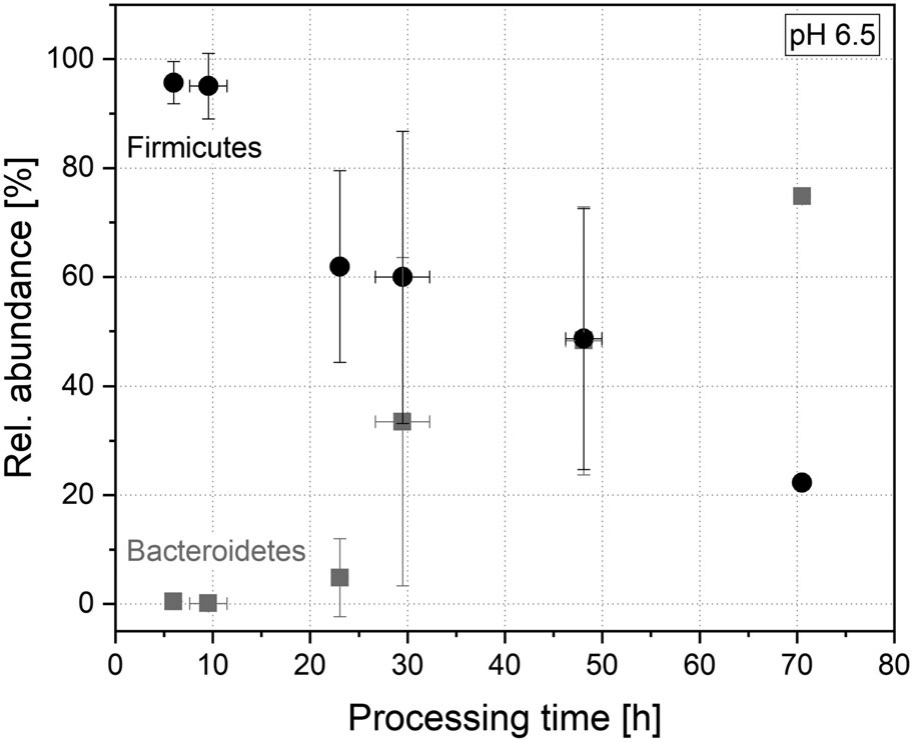
Rel. cum. abundance of the two major phyla Bacteroidetes (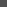) and Firmicutes (●) during re‐cultivation at pH 6.5.

Next to the metabolic behaviour of the system after drying, the microbial distribution was of interest. Therefore, the microbial composition was determined by sequencing 16S rRNA gene amplicons. When the microbiota cultivated at pH 6.5 was dried and re‐cultivated, significant changes in the abundance of the phyla during re‐cultivation can be observed and will be discussed in the following. The behaviour in the systems cultivated at pH 6.0 and 7.0 was comparable (data shown in Tables S1 and S2). For one of the major phylum, i.e., Bacteroidetes, a sharp decrease after drying to 0.46 ± 0.26% relative cumulative (rel. cum.) abundance was determined. Nevertheless, the abundance of the phylum could be restored and increased to 75.01 ± 0.79% after 71 h (Fig. 5). Obviously, Bacteroidetes are harmed through the cell harvesting and the drying process, but can be restored and re‐established.

**Fig. 6:**
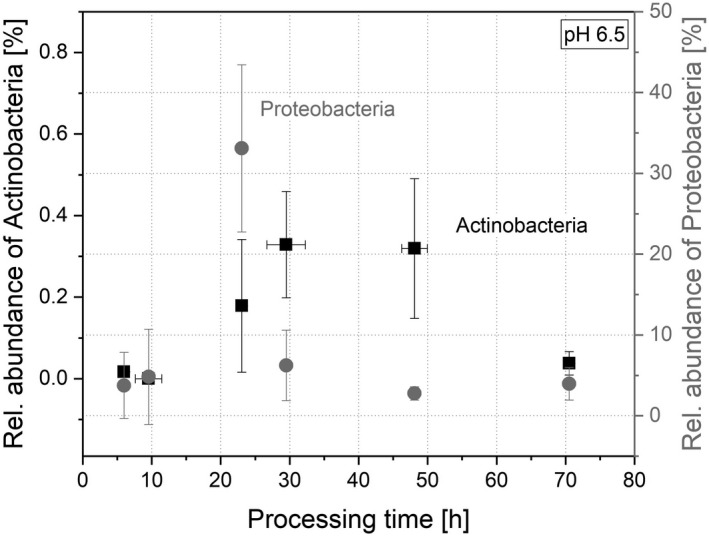
Rel. cum. abundance of Actinobacteria (■) and Proteobacteria (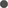) during re‐cultivation at pH 6.5.

In contrast, Firmicutes were not as sensitive after freeze–drying as Bacteroidetes. Their abundance was 95.69 ± 3.88% after 6 h (Fig. 5). During the further processing, the balance between Bacteroidetes and Firmicutes was re‐established and the abundance of Firmicutes settled to 22.27 ± 0.33% (71 h). Especially in the time range between 20 and 50 h of processing time, the ratio of Firmicutes and Bacteroidetes changed and resulted in a reversed ratio. The minor phylum Actinobacteria showed a similar behaviour as during cultivation as the phylum Firmicutes: After adaption, the abundance increased to 0.33 ± 0.13% (30 h), then decreased and plateaued to 0.04 ± 0.03% after 71 h (Fig. 6). The abundance of Proteobacteria progressed in a comparable way, but the peak in abundance occurred earlier. First, the abundance increased to 33.09 ± 10.35% (23 h), then decreased and plateaued to an abundance of 3.96 ± 2.02% (Fig. 6).

In contrast, the phylum of Verrucomicrobia could not be re‐established after preservation within the set limits (< 0.01% rel. cum. Abundance).

The ratio between Firmicutes and Bacteroidetes is a marker for human health. Here, during re‐cultivation at pH 6.5 a ratio of 0.30 was reached, indicating a healthy microbiota (Magne *et al*., 2020). Bircher *et al*. (2018) reached higher ratios for the re‐cultivation of donor 1 with 8.73 and 3.62, whereas donor 2 was in a comparable range of the Firmicutes to Bacteroidetes ratio (0.70) obtained here, indicating that the re‐established microbiota had a health indicating microbial composition.

**Fig. 7:**
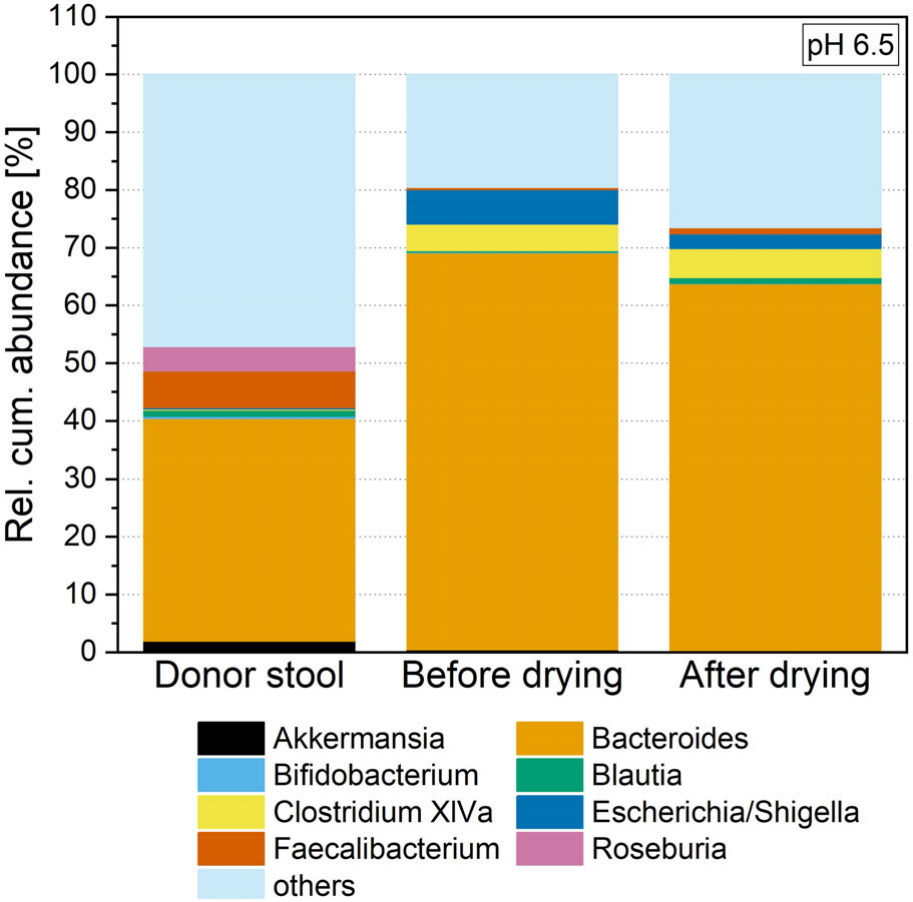
Rel. cum. abundance of genera in the donor stool and the stable system before and after drying at a cultivation pH of 6.5; for a better readability only genera discussed in this study are depicted, other genera are summed up as others.

In the human intestinal microbiome, several other genera are known as health markers, probiotics or other members important for diversity and composition. In Fig. 7, the abundance of *Akkermansia* (van Herreweghen *et al*., 2017), *Bacteroides* (Waidmann *et al*., 2003), *Bifidobacterium* (Saez‐Lara *et al*., 2015), *Blautia* (Shin *et al*., 2018), *Faecalibacterium* (Miquel *et al*., 2013; Cheema, 2019) and *Roseburia* (Siezen and Kleerebezem, 2011) as markers of intestinal health, as well as the abundance of the major genera *Clostridium* Cluster XIVa, *Escherichia* and *Shigella* (Siezen and Kleerebezem, 2011) is shown and discussed subsequently. All 30 further genera were summed up as ‘others’ to make the graph more readable. The diversity, measured by the Shannon effective index was also comparable at 29 (donor stool), 24 ± 1 before and 24 ± 6 after drying. *Akkermansia* and *Roseburia* were able to establish in the cultivation step prior to drying in small abundances (Haindl *et al*., 2021b). In the re‐cultivation step after drying they were hardly detectable (<0.01% abundance). Hence, it seemed as they are very sensitive to freeze–drying and not able to re‐grow in comparable numbers as before the drying step. The abundance of *Escherichia* and *Shigella* increased after re‐inoculation to 32.27 ± 9.70% (23 h) and decreased afterwards to 2.57 ± 1.42% after 71 h of processing time. Representatives of the genus *Escherichia* and *Shigella* are able to survive and even grow in an aerobic environment (Farmer and Jones, 1976). As contact with traces of oxygen during cell harvest and concentration prior to freeze–drying and re‐cultivation cannot be fully prevented, this genus was able to survive and re‐grow as the abundances of *Escherichia* and *Shigella* above show. The further genera *Bacteroides*, *Bifidobacterium*, *Blautia*, *Clostridium* Cluster XIVa and *Faecalibacterium* showed a similar behaviour after drying. All genera were present in low percentages after preservation, but could be re‐established. After 71 h of re‐cultivation an abundance of 63.83 ± 15.87% for *Bacteroides*, 0.03 ± 0.02% for *Bifidobacterium*, 1.00 ± 0.73% for *Blautia*, 5.05 ± 1.21% for *Clostridium* Cluster XIVa and 1.03 ± 0.35% for *Faecalibacterium* was detected. For these genera, the preservation process led to a decrease, but they were able to recover.

Other researches also investigated the abundance of butyrate‐producing bacteria *Faecalibacterium* and *Roseburia* after the preservation of *in vitro* microbiota through freezing (Bircher *et al*., 2018). They experienced a drop in the abundance of *Roseburia* after preservation. It seemed as *Roseburia* are sensitive to a preservation process of freezing and thawing as well as to freeze–drying. Possibly, not the removal of water is critical to this genus, but the freezing to low temperatures as it was done here as a part of freeze–drying as well as during cryopreservation performed by Bircher *et al*. (2018). Further, Bircher *et al*. (2018) measured a strongly impaired abundance of *Faecalibacterium* after cryopreservation. In this study, it was able to re‐establish. This may be due to different factors. On the one hand, the cultivation process prior to drying/ freezing differs between the two studies. *Faecalibacterium* showed impaired growth after freezing and thawing after the cultivation process applied by Bircher *et al*. (2018). Here, this genus was able to re‐establish after a continuous cultivation process and freeze–drying, although the survival of *Faecalibacterium* may be due to the different cultivation processes (continuous vs. batch in the re‐cultivation step). Further, the cells in this study did experience water removal through drying instead of thawing, which may have different effects on their ability to re‐establish. As the process conditions differ significantly (continuous vs. batch cultivation; different media; different preservation method) from the study of Bircher *et al*. (2018), we cannot conclude undoubtedly that freeze–drying itself is more appropriate for the genus of *Faecalibacterium* compared with freezing.

#### Diversity

Next to the microbial composition, the diversity of the microbiota is of importance when it comes to human health. In this study, diversity was measured with the criteria richness, representing the total number of OTUs in the community, and the Shannon effective index, which accounts for the evenness and abundance of species in the community. In the following, the data for re‐cultivation at pH 6.5 are discussed, and further data (pH 6.0 and 7.0) are shown in Tables S2 and S3. After drying and re‐inoculation, the richness in the system cultivated at pH 6.5 decreased to 47 ± 12 but increased afterwards to 104 ± 11 (71 h processing time). Compared with the system prior to freeze–drying, the richness is comparable (99 ± 7). Further, the Shannon effective index was calculated. After inoculation, the evenness and abundance of different species in the system re‐cultivated at pH 6.5 was very low at a value of 3 ± 1 (6 h). As the contribution of phyla and genera already showed, the species were able to re‐establish and consequently, the Shannon effective index increased to 8 ± 3 (23 h) and finally to 24 ± 6 (71 h). Compared with the stable system before preservation, the Shannon effective index was in the same range (24 ± 1 prior to freeze–drying). As a result, neither richness nor Shannon effective index is influenced by the drying process applied in this study and therefore indicates a healthy and diverse system. Contrary results where reached by Bircher *et al*. (2018), who detected a lower diversity in the re‐cultivated system. This gives the hint, as if freeze–drying is able to restore the diversity, whereas a preservation through freezing might lead to a decrease. Nevertheless, here also the different cultivation techniques have to be considered. Consequently, no clear statement can be made regarding the question of which preservation method being more appropriate.

### Ability to re‐establish after the drying step: comparison of the pre‐ and post‐drying system

To investigate the influence of the ability to re‐establish after the drying process, the values of the stable system prior to freeze–drying were compared with the characteristics of the re‐established system (data in Table S3). The open question was, whether different or comparable systems were generated after drying to reveal the impact of the drying process itself and the effect of drying stress on the individual phyla and genera.

For the *in vitro* microbiota cultivated at pH 6.5, only few significant differences based on a p‐value of 0.05 were detected. For a number of aerobic and anaerobic CFUs, no differences after re‐cultivation were detectable. Further, the microbial composition showed no difference in the abundance of the five phyla. Also, on genera level no differences between the stable systems before and after drying occurred. The α‐diversity, richness and Shannon effective index were similar as well. As discussed above, the drying step led to a significant decrease of the cell count. Nevertheless, the system was able to re‐establish. Hence, number and composition of microorganisms were influenced by freeze–drying in a reversible way and the system was able to re‐establish completely regarding these factors. Contrary, differences in the production of metabolites were detected. After cultivation at pH 6.5, the concentration of acetate increased after drying from 3.82 ± 0.14 mg ml^−1^ (stable system prior to drying) to 4.35 ± 0.26 mg ml^−1^ after 71 h of re‐cultivation. By contrast, the concentration of propionate and iso‐valerate was decreased in the broth after re‐cultivation compared with the stable system before drying. Propionate decreased from a concentration of 2.94 ± 0.13 mg ml^−1^ of about 14% to 2.51 ± 0.19 mg ml^−1^ (71 h of re‐cultivation). Iso‐valerate showed a decrease of about 58% from 0.36 ± 0.08 mg ml^−1^ (stable system prior to drying) to 0.16 ± 0.06 mg ml^−1^ in the re‐cultivated broth. The concentrations of butyrate showed no significant differences at pH 6.5. In total, the metabolism of the re‐established microbiota, measured by the total concentration of SCFAs was not affected irreversible by the drying step. The systems cultivated at pH 6.0 and 7.0 showed comparable behaviour with only significant changes regarding the metabolism in the established systems pre and post drying. For pH 6.0, all investigated metabolites as well as the overall concentration of SCFAs differed: As already observed at pH 6.5, the concentration of acetate was increased after drying. Here, also propionate and iso‐valerate were decreased, as well as butyrate and the overall concentration (Table S3). For a re‐cultivation at pH 7.0, only the concentrations of butyrate and iso‐valerate were decreased. The changes in metabolism after drying observed in this study are partly significant, but do not automatically indicate an altered microbial composition as the distribution of phyla before and after freeze–drying showed. Hence, the alterations are probably due to the natural dynamics of the microbiota and do not indicate a change induced completely by the drying process (Gerber, 2014). In contrast, when preserving the microbiota through freezing, a lowered metabolism was observed (Bircher *et al*., 2018). Further, the concentration of acetate, propionate and butyrate decreased after cryopreservation. Nevertheless, this trend has to be proven urgently with more experiments and further studies, before a clear recommendation for the preservation process can be made.

### Influence of the cultivation pH: impact on the re‐established system

For the drying of probiotic single cell cultures, the influence of the cultivation pH was already investigated and shown to have a major impact (Bauer *et al*., 2012). Further, the impact during the establishment of an *in vitro* microbiota was shown in former studies (Haindl *et al*., 2021b). In this study, the *in vitro* microbiota was cultivated prior to and after the drying process at three different physiological pH values (pH 6.0, 6.5, 7.0). The open question was, whether one of the pH values has a positive effect on drying resistance and the re‐established system and creates a system after lyophilization that is most similar to the donor stool. In the following, the influence of the cultivation pH on the re‐established system will be evaluated. Therefore, we compared the values of all characteristics in the stable system after a cultivation time of at least 70 h based on a one‐way ANOVA (p ≤ 0.05) followed by a Tukey *post hoc* analysis. The data are presented in Table S3.

For cell count, no significant difference occurred for either aerobic or anaerobic microorganisms. All of the three investigated pH values created an *in vitro* system with a comparable number of cells. Otherwise, their metabolism, measured by the SCFAs showed some pH dependent differences. In total, the sum of SCFAs showed a peak in concentration at pH 6.5. It seemed as at this pH value a more active metabolism was able to recover. This peak in SCFAs was due to the composition of the major acids. The peak at pH 6.5 was also determined for the concentration of butyrate. The concentration of acetate was not influenced by the cultivation pH. For propionate, a higher concentration was metabolized at pH 7.0, whereas iso‐valerate showed a contrary behaviour with the lowest concentration at pH 7.0. Consequently, the peak in metabolism, measured by the sum of all SCFAs is probably due to a high abundance of butyrate‐producing bacteria in the experiment conducted at pH 6.5. Regarding the microbial composition based on phyla level in the three systems after drying, only Bacteroidetes and Firmicutes showed a pH‐dependency in their abundance. While the abundance of Bacteroidetes increased with the pH value, the abundance of Firmicutes, in contrast, decreased. This effect was already shown during cultivation before drying (Haindl *et al*., 2021b), but did not explain the peak in butyrate production after drying during re‐cultivation. On genera level, *Faecalibacterium, Clostridium* Cluster XIVa, *Blautia* and *Roseburia* are the main butyrate‐producing bacteria in the microbiota (Bircher *et al*., 2018). Based on genera level, only *Faecalibacterium* showed a pH‐dependent abundance. As already described above, the abundance of *Roseburia* after 70 h of re‐cultivation was not detectable anymore within the set limits. Nevertheless, the abundance of *Faecalibacterium* after drying was between 0.01 ± 0.005% (pH 6.0), 0.01 ± 0.01% (pH 7.0) and 1.03 ± 0.35% (pH 6.5) and therefore explained the peak in butyrate‐production at pH 6.5. *Clostridium* Cluster XIVa, *Blautia and* Roseburia show no dependency in their abundance based on the cultivation pH value. *Blautia* shows a slight trend to higher abundances at higher cultivation pH values, but this trend is not significant. Consequently, this cannot explain the peak in butyrate production but may support the higher concentrations at pH 6.5

Overall, the variation of cultivation pH value led to different systems. Their behaviour was, as discussed above and in further studies similar to the results before drying (Haindl *et al*., 2021b). When comparing the re‐established systems after 70 h, a peak in metabolism due to a higher abundance of butyrate‐producing *Faecalibacterium* was observed. In total, the re‐cultivation pH does only affect the metabolism as well as the abundance of Firmicutes, Bacteroidetes and *Faecalibacterium*.

### Influence of the entire process chain: comparison of the initial faeces, pre‐ and post‐drying system

Former chapters above have already shown that the drying step itself does only have a small influence on the metabolism when comparing the pre‐ and post‐drying system. The pH value during re‐cultivation was found to influence the metabolism, especially butyrate production, as well as the abundance of the major phyla Bacteroidetes and Firmicutes. When preserving the microbiota, not only single process steps, but the overall process chain is of importance. In the following, the microbial composition and diversity of the initial faeces will be compared with the pre‐ as well as the post‐drying system. These data are presented in in Table S3.

**Fig. 8:**
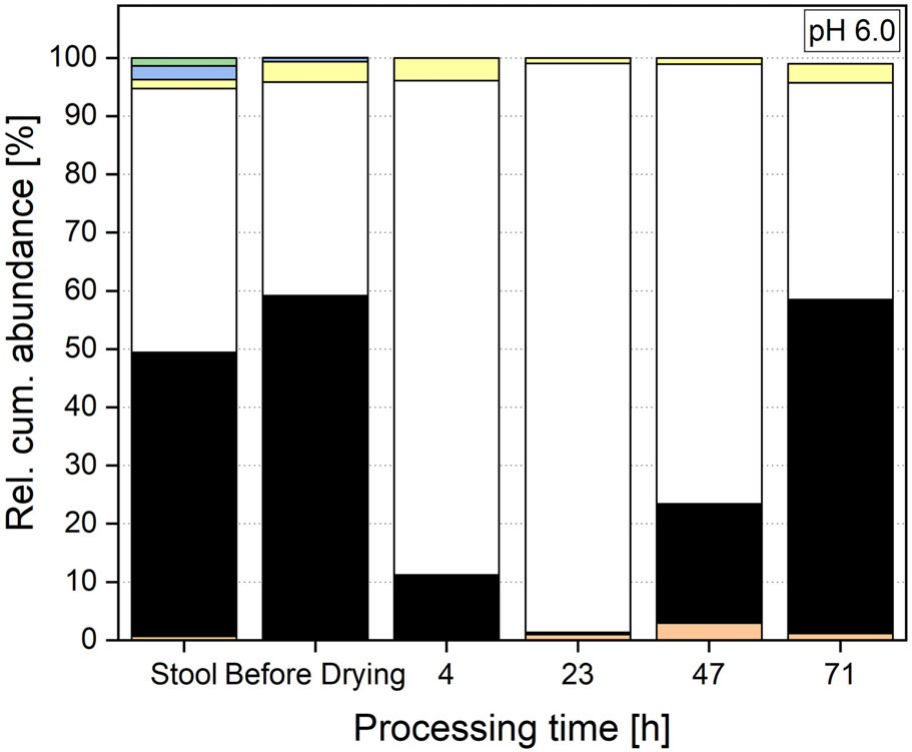
Relative cumulative abundance of phyla in the donor stool, stable system before drying and at several processing points during re‐cultivation at a cultivation pH of 6.0.

**Fig. 9:**
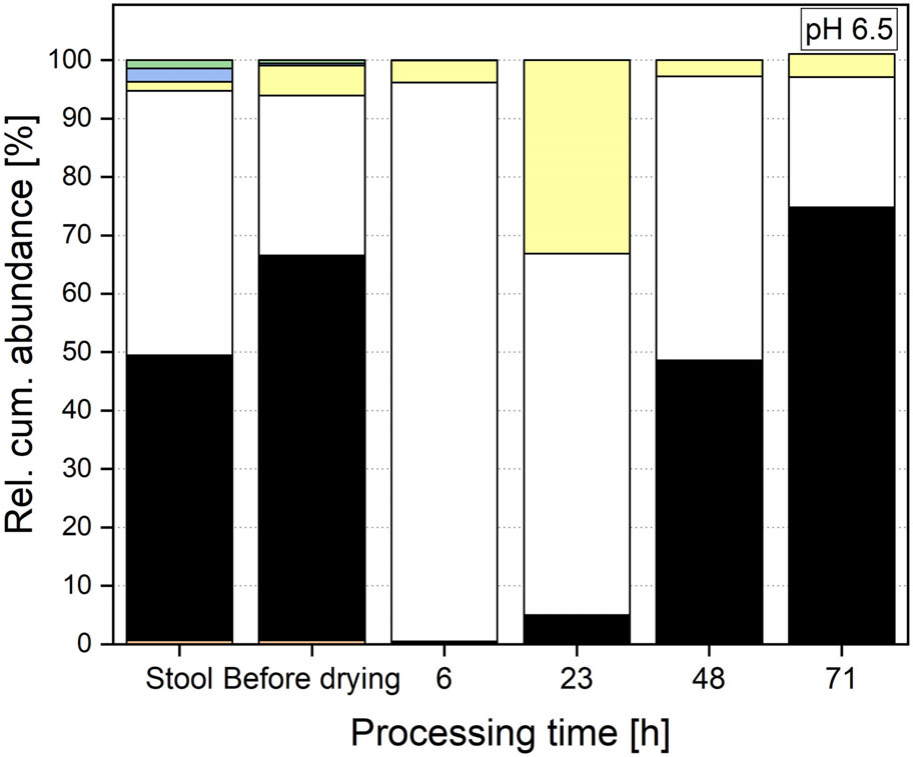
Relative cumulative abundance of phyla in the donor stool, stable system before drying and at several processing points during re‐cultivation at a cultivation pH of 6.5.

**Fig. 10:**
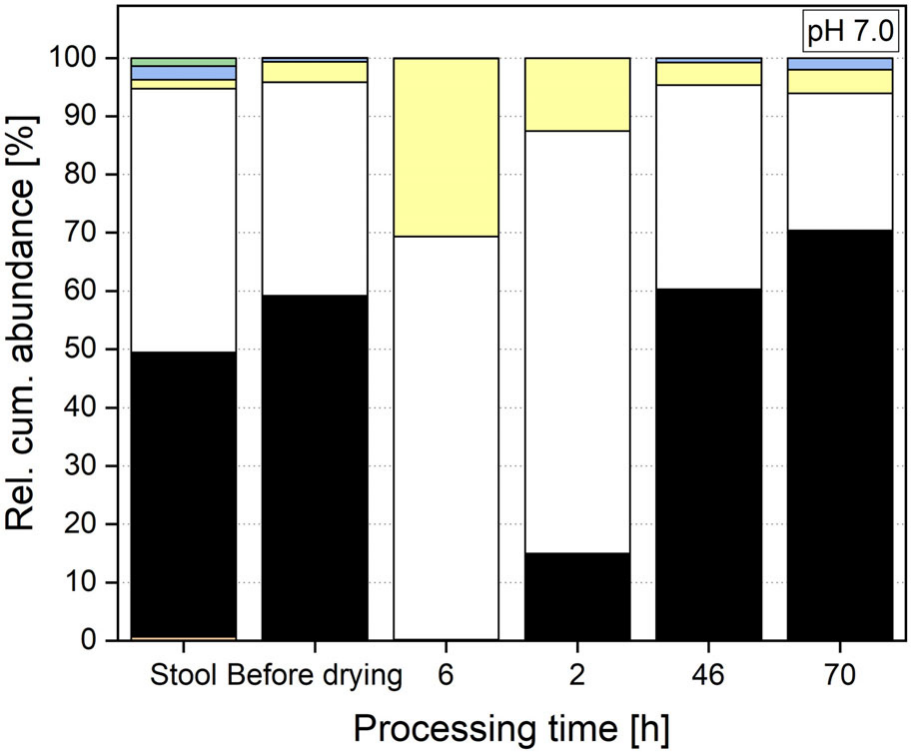
Relative cumulative abundance of phyla in the donor stool, stable system before drying and at several processing points during re‐cultivation at a cultivation pH of 7.0.

Comparing the microbial diversity of the faeces with the post‐drying system, no significant differences can be observed for either richness or the Shannon effective index. The richness in the post‐drying, re‐cultivated system (e.g., 104 ± 11 for pH 6.5) was comparable with the donor stool with a richness of 99. The Shannon effective index, which was 29 in the stool, was in the same range as in the re‐cultivated system (e.g., 24 ± 6 for pH 6.5) and did not differ for any of the investigated pH values based on a p‐value of 0.05. These results are different from other studies where the *in vitro* microbiota was preserved through freezing (Bircher *et al*., 2018). Next to diversity, the microbial composition is important and was investigated and faeces compared with the post‐drying system as shown in Figs 8, 9, 10. Here, no differences for the minor phyla Actinobacteria, Proteobacteria and Verrucomicrobia were detected when comparing faeces with all three post‐drying systems. For Firmicutes, the abundance in the stool was significantly higher. In contrast, the abundance of Bacteroidetes in the stool was lower compared with pH 6.5 and 7.0, whereas the abundances at pH 6.0 (49.11 ± 14.45%) and the donor stool (48.79%) did not differ significantly. These results originate and proceed from the cultivation step. There, also the abundances of Firmicutes were decreased and Bacteroidetes increased compared with the donor stool (Haindl *et al*., 2020).

Next to the abundance, the ratio of Firmicutes to Bacteroidetes is of interest. In the initial donor stool, the ratio was 0.93. Regarding the distribution of phyla, a cultivation pH of 6.0 creates a system most similar to the donor sample during re‐cultivation. At pH 6.0, the ratio of Firmicutes to Bacteroidetes is 0.75, compared with 0.30 (pH 6.5) and 0.34 (pH 7.0). When looking at the microbial composition at genera level, different observation for the selected genera can be made. For *Bifidobacterium*, *Blautia*, *Escherichia* and *Shigella*, no significant differences in abundances between the donor stool and the re‐cultivated microbiota can be seen. These genera seemed to be not sensitive to the entire preservation process and can be conserved in their initial abundances. *Bacteroides* and *Clostridium* Cluster XIVa showed increased abundances in the stable system after drying compared with the donor stool for the cultivation at pH 7.0 and 6.5. For a pH value of 6.0, no significant differences in the abundances of these two genera compared with the donor stool were detected. These two genera had higher tolerances regarding the preservation process when cultivated at higher pH values (6.5 and 7.0) and therefore were abundant in higher values. Indeed, a cultivation at pH 6.0 led to a process resistance that created an abundance after the whole process comparable with the initial faeces. However, these altered abundances did not occur during re‐cultivation, but appeared already during cultivation and therefore before drying (Haindl *et al*., 2021b). The drying process itself did not change the composition additionally. As already discussed above, *Akkermansia* and *Roseburia* were very sensitive to the preservation process and only abundant in hardly detectable abundances in the re‐cultivated system (e.g., *Akkermansia* showed an abundance of 0.001 ± 0.01% at system pH 6.0 compared with 1.98% in the donor stool). Also, the abundance of *Faecalibacterium* was lowered (e.g., 1.03 ± 0.5% after re‐cultivation at pH 6.5) compared with the donor stool (6.39%). Consequently, these compositional changes resulted from the cultivation process that took place before drying.

As already investigated in previous studies, the cultivation itself and further the applied conditions during cultivation had a major impact on the composition of the *in vitro* microbiota (Haindl *et al*., 2020, 2021b). The results discussed in this chapter proceed over the whole preservation process as they show the same differences between the re‐cultivated microbiota and the initial donor stool system. As only slight differences in the metabolism between the cultivation systems prior and post drying were detected, the changes in the microbial composition in the preserved *in vitro* microbiota are probably due to the cultivation step before drying instead of the drying step itself. Concluding from that and summing up the results from above, the cultivation step had a higher impact on the entire process chain than the drying step. Further, the cultivation pH before freeze–drying was the value that may be adjusted to reach a high abundance, diversity and richness comparable with the donor stool. Consequently, major alterations in the *in vitro* microbiota result from the cultivation step prior to drying. The drying itself did not cause any significant changes anymore.

## Conclusions

In the current study, the influence of an entire preservation process on the survival, vitality, viability and ability to re‐grow of a human intestinal microbiota was investigated. Therefore, the impact of the cultivation pre‐ and post‐drying, the drying step itself on cell count, metabolism, microbial composition, diversity as well as the re‐formation of a stable system was determined.

After drying, the moisture in the received powder indicated a stable and storable product but showed a low survival rate of the cells. Nevertheless, it was proven that the system was able to re‐establish and re‐grow a healthy system within 24–70 h. Cell count, microbial composition as well as diversity did not differ significantly for all investigated pH values compared with the cultivation system prior to drying. Metabolism after drying showed slight changes in the concentration of SCFAs, but these alterations are more due to natural dynamics of the microbiota and do not indicate a change induced by the drying‐process.

The aim of this study was to identify process steps that lead to major alterations of the *in vitro* system compared with the initial donor stool. When comparing the *in vitro* systems prior and post drying, it is clearly visible that hardly any changes occur during freeze–drying and therefore prior to drying, already during cultivation. The drying step influenced cell count and microbial composition significantly. Nevertheless, these effects were reversible and vanished during re‐cultivation. The cultivation step, especially the cultivation pH value, had a major impact and offered potential for optimization of the entire preservation process. We detected different influences of the cultivation pH on characteristics of the re‐established system. For cell count as well as for diversity, measured by richness and the Shannon effective index, no pH dependent influence was observed. Additionally, all cultivation pH values resulted in cells with a healthy and working metabolism. Nevertheless, a cultivation pH of 6.5 provided the most comparable, healthy characteristics in metabolism. Regarding the distribution of phyla and genera, a cultivation pH of 6.0 led to a composition that is closer to the initial system in the donor stool than at higher pH values. Consequently, for the preservation of an *in vitro* microbiota, we recommend lower physiological pH values between 6.0 and 6.5.

The application of *in vitro* infusion material instead of a traditional FMT had already been studied (Garborg, 2015). As we had proven that a dried microbiota does technically not differ significantly from that, it gives the hint that a freeze–dried *in vitro* microbiota might also be a possibility of a successful treatment. Nevertheless, it urgently needs the investigation of further stool samples as well as *in vivo* studies, since it is not clear whether the impact on patients’ recovery of cultivated bacteria is the same as FMT itself.

## Experimental procedures

### Process Flow

**Fig. 11:**
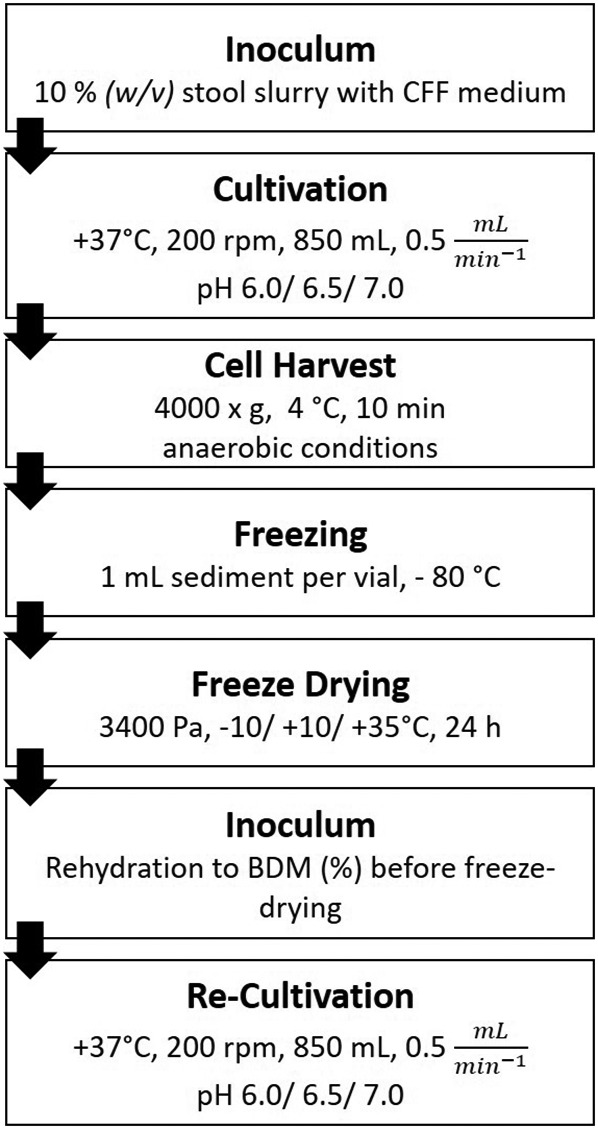
Process scheme applied in this study.

Stool from a healthy donor was purified and used as the inoculum for an *in vitro* cultivation system (Fig. 11). The microbiota was cultivated at either pH 6.0, 6.5 or 7.0 with the conditions described below. After 120 h of cultivation, the broth was harvested, concentrated by centrifugation (Allegra X15R, Beckmann Coulter, Brea, CA, USA; 4.000 x g, +4°C, 10 min) and afterwards 1 ml of the sediment each was dosed into freeze–drying glass vials and stored at −80°C. After freeze–drying, the powder was rehydrated to its initial biological dry matter (BDM, %) and used as an inoculum for the re‐cultivation process. Re‐cultivation was performed to assess viability, vitality and ability of the dried microbiota to regrow in a controlled *in vitro* environment. For re‐cultivation, the same pH value as during cultivation was used. Each cultivation run and freeze–drying was done in triplicate. Further, all analyses were executed at least three times for each experiment.

### Donor stool

Former studies had already investigated the influence of different stool samples and revealed only a minor influence. Consequently, this study was conducted with stool from one healthy, female donor and one defecation (Haindl *et al*., 2020). The donor was chosen according to the criteria important for FMT (Terveer *et al*., 2017) and tested prior to the experiments for bacterial, viral and eukaryotic pathogens by the Institute for Medical Microbiology and Hygiene, University of Regensburg to prevent the transfer of diseases. The stool was obtained in‐house and stored immediately at −80°C until use. The donor was Caucasian, ate a Western diet, had a body mass index of 21 and was 25 years old. The last antibiotic treatment was over 12 months ago. The abundance of phyla in the stool was as follows: 0.65% Actinobacteria, 48.79% Bacteroidetes, 45.28% Firmicutes, 1.59% Proteobacteria, 2.30% Verrucomicrobia and 1.39% of unknown phyla. The richness was 99 and the Shannon effective index 29. Consequently, this donor hosted a microbiota considered as healthy regarding microbial composition, richness and diversity (Rinninella *et al*., 2019).

### *In vitro* cultivation system

Prior to and after the freeze–drying step, an *in vitro* cultivation of the microbiota was performed as already established in former studies (Haindl *et al*., 2021a). The cultivation system was used either to obtain samples for the drying experiments or to test viability, vitality and the ability to re‐establish a stable system after drying. As medium for the cultivation as well as for the preparation of inoculum, a so‐called continuous flow fermentation (CFF) medium was used, which was already described in previous studies (Haindl *et al*., 2021a). The preparation of inoculum for the cultivation step prior to drying was already described previously (Haindl *et al*., 2021a).

After drying, the powder was rehydrated with sterile bi‐distilled water to its initial biological dry matter (after cell harvest and concentration, before drying). The obtained solution was then joint with 120 ml fresh CFF medium and served as the inoculum.

During processing, samples were collected by pumping broth anaerobically into prepared sample tubes (Greiner Bio‐One, Sigma Aldrich, St Louis, USA). The broth was either used immediately for further analysis (cell count, SCFAs) or stored at −80°C until analysis (16S rRNA gene amplicon sequencing).

### Freeze–drying process

Prior to the drying process, the samples were frozen at −80°C for at least 24 h. The freeze–drying process used in this study was already established in a previous work based on the drying of a probiotic strain from the human intestinal microbiota (Haindl *et al*., 2020). The process was conducted in a Delta 1–24 LSC pilot plant dryer (Christ GmbH, Osterode, Germany). The chamber pressure was set at a constant 3700 Pa through the whole processing time, while the shelf temperature rose in a stepwise manner: −10°C for 12 h, +10°C for 6 h and +35°C for 6 h. Consequently, the whole drying process took 24 h.

### Analysis for residual moisture content and water activity

The residual moisture content and water activity were determined after each drying process. The residual moisture content was measured by using a CEM Smart Turbo™ 5 (CEM Corporation, Kamp‐Lintfort, Germany) at a maximum sample temperature of +80°C and 45% power input. The results were double‐checked regularly with Karl Fischer titration using TitroLine KF (Mettler‐Toledo GmbH, Schwerzenbach, Germany).

Water activity a_w_ was measured using an Aw Sprint TH‐500 (Novasina, Lachen, Switzerland).

### Analysis of cell count

Cell counts were analysed for anaerobic and aerobic cells separately. After collection, the samples were diluted with 0.25 strength Ringer’s solution and then plated on either Wilkins‐Chalgren Anaerobe agar plates (anaerobic cell count) or Plate Count agar plates (facultative aerobic cell count). The plates were incubated for 48 h at +37°C either aerobically or anaerobically in an anaerobic chamber heated to +37°C. For data evaluation, only plates with 30–300 colonies were considered and the number of colony‐forming units N per ml of sample (CFU ml^−1^) was calculated according to the following equation:

(1) $$N=\frac{c}{n_{1}+\left(0.1\;\cdot \;n_{2}\right)}$$

Here, *c* is the sum of colonies of the subsequent dilutions; *n_1_* is the number of colonies in the less diluted cell suspensions and *n_2_* is the number of colonies in the more diluted solution.

### Analysis of short‐chain fatty acids by High‐Performance Liquid Chromatography

The metabolic behaviour of the *in vitro* microbiota was revealed by analysing the main short‐chain fatty acids (SCFAs) acetate, propionate, butyrate and iso‐valerate by a high‐performance liquid chromatography (HPLC; Agilent Technologies, Santa Clara, USA) system equipped with an Aminex HPXH‐87H ion exclusion column (Bio‐Rad Laboratories, Hercules, USA) and a G1362A refractive index detector (Agilent Technologies, Santa Clara, USA). The injection volume was set to 20–100 µl and separation was performed with 0.0005 mol l^−1^ H_2_SO_4_ at a flow rate of 0.45 ml min^−1^. Prior to the measurement, the samples got centrifuged (Hermel‐Z233 M‐2, Hermle Labortechnik GmbH, Wehingen, Germany; 6000 *g*, +20°C, 30 min) and the supernatant filtrated with a 0.22 µm sterile filter. SCFAs were identified and quantified using external standards (Sigma Aldrich, Saint Louis, USA) and the software Agilent ChemStation Instrument 1 Offline (Agilent Technologies, Santa Clara, USA).

### Microbiota profiling with 16S rRNA gene amplicon sequencing

The microbial community, richness and diversity were analysed at several time points during cultivation. For this purpose, the V3/V4 region of 16S rRNA was sequenced by High‐throughput 16S rRNA Gene Amplicon Sequencing, which was performed by the Microbiome Core Facility, ZIEL, TU Munich, according to a protocol previously described and used in earlier studies (Reitmeier *et al*., 2020; Haindl *et al*., 2021a,b).

### Statistical analysis

All cultivations, dryings and further analyses were repeated at least in triplicate. The mean values are shown as the arithmetic mean $\bar{x}$ of the number *n* of all samples *x_i_*. The distribution of the values was calculated from the standard deviation *s* due to the random error. All graphs show the arithmetic means ± standard deviations. Statistical significance was tested using a one‐way ANOVA (*P* ≤ 0.05) followed by a Tukey *post hoc* analysis with the software *OriginPro 2019* (OriginLab Corporation, Northampton, USA).

## Conflict of interest

The authors declare that they have no conflict of interest.

## Author contributions

R.H. and U.K. conceived of and designed the experiments. L.T. and R.H. performed the experiments and analysis. R.H. prepared the figures, made statistical analysis and wrote the manuscript. L.T., R.H. and U.K. revised the manuscript.

## Informed consent status

Informed consent was obtained from all subjects involved in the study.

## Supporting information


**Table S1**. Evolvement of all investigated values during re‐cultivation at pH 6.0.
**Table S2**. Evolvement of all investigated values during re‐cultivation at pH 7.0.
**Table S3**. Summary and comparison of all investigated values of the stool sample, in the stable system before and after drying depending on the cultivation pH value.

## Data Availability

Raw sequencing data are available at the Sequence Read Archive under the accession number PRJNA766833.
