# Supplementary material for: Preservation by lyophilization of a human intestinal microbiota: influence of the cultivation pH on the drying outcome and re‐establishment ability
Source: Microb Biotechnol. 2022 Feb 6;15(3):886–900. doi: 10.1111/1751-7915.14007 (PMC8913864; doi:10.1111/1751-7915.14007)
Supplement: Supplementary file 1 — Table S1. Evolvement of all investigated values during re‐cultivation at pH 6.0. Table S2. Evolvement of all investigated values during re‐cultivation at pH 7.0. Table S3. Summary and comparison of all investigated values of the stool sample, in the stable system before and after drying depending on the cultivation pH value. [file MBT2-15-886-s001.docx]

# Supplementary Data

Table 2: Evolvement of all investigated values during re-cultivation at pH 6.0

|  | **Processing time [h]** | | | | | |
| --- | --- | --- | --- | --- | --- | --- |
| **Investigated value** | *4* | *12* | *23* | *34* | *47* | *71* |
| CFU aerob [10^9^ CFU mL^-1^] | - | - | 2 ± 0.3 | - | 2 ± 1 | 0.2 ± 0.1 |
| CFU anaerob [10^9^ CFU mL^-1^] | - | - | 3 ± 1 | - | 9 ± 7 | 7 ± 1 |
| Σ SCFA [mg mL^-1^] | 0.19 ± 0.12 | 1.69 ± 0.47 | 2.64 ± 0.46 | 3.44 ± 0.50 | 5.51 ± 1.61 | 7.73 ± 1.09 |
| Acetate [mg mL^-1^] | 0.13 ± 0.08 | 1.13 ± 0.13 | 1.93 ± 0.12 | 2.38 ± 0.33 | 3.29 ± 0.63 | 4.39 ± 0.34 |
| Butyrate [mg mL^-1^] | 0.01 ± 0.02 | 0.30 ± 0.21 | 0.38 ± 0.20 | 0.71 ± 0.12 | 1.05 ± 0.12 | 0.97 ± 0.05 |
| Propionate [mg mL^-1^] | 0.02 ± 0.01 | 0.05 ± 0.02 | 0.13 ± 0.07 | 0.17 ± 0.03 | 0.95 ± 0.80 | 2.13 ± 0.62 |
| Iso-valerate [mg mL^-1^] | 0.02 ± 0.01 | 0.20 ± 0.10 | 0.20 ± 0.06 | 0.18 ± 0.01 | 0.22 ± 0.06 | 0.24 ± 0.08 |
| Richness [-] | 73 ± 28 | 30 ± 18 | 35 ± 16 | 40 ± 14 | 60 ± 21 | 75 ± 23 |
| Shannon effective [-] | 7 ± 4 | 5 ± 1 | 4 ± 2 | 8 ± 1 | 15 ± 7 | 25 ± 8 |
| Actinobacteria [%] | 0.02 ± 0.02 | 0.36 ± 0.62 | 0.96 ± 1.07 | 2.52 ± 0.63 | 2.92 ± 0.79 | 1.18 ± 0.92 |
| Bacteroidetes [%] | 11.17 ± 8.46 | 0.52 ± 0.79 | 0.38 ± 0.25 | 1.46 ± 0.81 | 20.46 ± 18.56 | 57.32 ± 3.70 |
| Firmicutes [%] | 84.90 ± 11.18 | 98.22 ± 1.07 | 97.70 ± 1.41 | 95.20 ± 2.03 | 75.54 ± 18.60 | 37.23 ± 1.30 |
| Proteobacteria [%] | 3.88 ± 2.81 | 0.89 ± 0.89 | 0.95 ± 0.53 | 0.82 ± 0.73 | 1.08 ± 0.83 | 3.27 ± 2.95 |
| Verrucomicrobia [%] | < 0.01 | < 0.01 | < 0.01 | < 0.01 | < 0.01 | < 0.01 |
| *Akkermansia* [%] | < 0.01 | < 0.01 | < 0.01 | < 0.01 | < 0.01 | < 0.01 |
| *Bacteroides* [%] | 8.86 ± 6.77 | 0.50 ± 0.80 | 0.35 ± 0.19 | 1.45 ± 0.81 | 20.42 ± 18.49 | 48.70 ± 14.23 |
| *Bifidobacteria* [%] | 0.02 ± 0.02 | 0.36 ± 0.62 | 0.96 ± 1.07 | 2.52 ± 0.63 | 2.78 ± 0.72 | 1.16 ± 0.93 |
| *Blautia* [%] | 0.28 ± 0.20 | < 0.01 | < 0.01 | 0.08 ± 0.08 | 2.29 ± 1.86 | 4.45 ± 3.88 |
| *Faecalibacterium* [%] | 0.95 ± 0.73 | < 0.01 | < 0.01 | < 0.01 | < 0.01 | < 0.01 |
| *Roseburia* [%] | < 0.01 | < 0.01 | < 0.01 | < 0.01 | < 0.01 | < 0.01 |
| *Clostridium* Cluster XIVa [%] | 0.38 ± 0.30 | < 0.01 | < 0.01 | < 0.01 | 0.14 ± 0.22 | 2.62 ± 1.80 |
| *Escherichia/ Shigella* [%] | 3.80 ± 2.79 | 0.85 ± 0.92 | 0.92 ± 0.50 | 0.78 ± 0.68 | 0.80 ± 0.58 | 2.40 ± 2.36 |

Table 3: Evolvement of all investigated values during re-cultivation at pH 7.0

|  | **Processing time [h]** | | | | | |
| --- | --- | --- | --- | --- | --- | --- |
| **Investigated value** | *6* | *10* | *22* | *32* | *46* | *70* |
| CFU aerob [10^9^ CFU mL^-1^] | - | - | 9 ± 0 | - | 2 ± 2 | 0.5 ± 0.2 |
| CFU anaerob [10^9^ CFU mL^-1^] | - | - | 6 ± 6 | - | 8 ± 9 | 6 ± 2 |
| Σ SCFA [mg mL^-1^] | 0.31 ± 0.07 | 1.31 ± 0.99 | 3.88 ± 0.48 | 5.84 ± 0.99 | 7.39 ± 1.31 | 8.35 ± 1.11 |
| Acetate [mg mL^-1^] | 0.21 ± 0.06 | 0.85 ± 0.45 | 2.40 ± 0.22 | 3.83 ± 0.19 | 4.77 ± 0.48 | 4.64 ± 0.48 |
| Butyrate [mg mL^-1^] | 0.01 ± 0.00 | 0.23 ± 0.44 | 1.08 ± 0.15 | 1.05 ± 0.25 | 0.94 ± 0.12 | 0.90 ± 0.06 |
| Propionate [mg mL^-1^] | 0.06 ± 0.00 | 0.10 ± 0.08 | 0.23 ± 0.09 | 0.77 ± 0.42 | 1.55 ± 0.68 | 2.67 ± 0.53 |
| Iso-valerate [mg mL^-1^] | 0.03 ± 0.01 | 0.12 ± 0.02 | 0.17 ± 0.02 | 0.19 ± 0.13 | 0.12 ± 0.04 | 0.13 ± 0.04 |
| Richness [-] | 34 ± 21 | 32 ± 18 | 39 ± 18 | 58 ± 13 | 64 ± 18 | 68 ± 25 |
| Shannon effective [-] | 3 ± 2 | 4 ± 2 | 9 ± 4 | 14 ± 2 | 17 ± 4 | 18 ± 6 |
| Actinobacteria [%] | < 0.01 | < 0.01 | < 0.01 | 0.18 ± 0.35 | 0.11 ± 0.19 | 0.02 ± 0.02 |
| Bacteroidetes [%] | 0.18 ± 0.12 | 0.13 ± 0.08 | 14.95 ± 21.12 | 53.77 ± 22.31 | 60.19 ± 13.13 | 70.35 ± 5.79 |
| Firmicutes [%] | 69.14 ± 35.95 | 77.89 ± 7.96 | 72.52 ± 16.01 | 40.29 ± 20.55 | 35.06 ± 11.21 | 23.61 ± 6.16 |
| Proteobacteria [%] | 30.63 ± 35.82 | 21.93 ± 7.97 | 12.53 ± 5.75 | 5.73 ± 1.63 | 3.89 ± 2.08 | 4.05 ± 1.84 |
| Verrucomicrobia [%] | 0.03 ± 0.01 | < 0.01 | < 0.01 | < 0.01 | 0.75 ± 1.26 | 1.98 ± 3.36 |
| *Akkermansia* [%] | 0.03 ± 0.01 | < 0.01 | < 0.01 | < 0.01 | 0.81 ± 1.22 | 2.00 ± 3.34 |
| *Bacteroides* [%] | 0.16 ± 0.11 | 0.12 ± 0.08 | 11.93 ± 16.12 | 43.29 ± 18.45 | 60.12 ± 18.14 | 70.79 ± 9.97 |
| *Bifidobacteria* [%] | < 0.01 | < 0.01 | < 0.01 | 0.18 ± 0.45 | 0.11 ± 0.19 | 0.02 ± 0.02 |
| *Blautia* [%] | < 0.01 | < 0.01 | < 0.01 | 0.66 ± 1.01 | 0.80 ± 0.95 | 0.26 ± 0.27 |
| *Faecalibacterium* [%] | 0.01 ± 0.00 | 0.02 ± 0.03 | 0.19 ± 0.33 | < 0.01 | < 0.01 | < 0.01 |
| *Roseburia* [%] | < 0.01 | < 0.01 | < 0.01 | < 0.01 | < 0.01 | < 0.01 |
| *Clostridium* Cluster XIVa [%] | 0.04 ± 0.01 | 0.02 ± 0.02 | 1.05 ± 1.76 | 2.78 ± 3.80 | 4.87 ± 2.66 | 4.21 ± 1.78 |
| *Escherichia/ Shigella* [%] | 29.64 ± 34.42 | 21.61 ± 7.52 | 10.58 ± 8.11 | 5.67 ± 2.36 | 2.70 ± 2.52 | 3.24 ± 2.47 |

Table 4: Summary and comparison of all investigated values of the stool sample, in the stable system before and after drying depending on the cultivation pH value

| **Investigated value** | **Stool** | **Before drying, after cultivation (120 h) and concentration** | | | **After drying in the re-established system (>70 h cultivation time)** | | | |
| --- | --- | --- | --- | --- | --- | --- | --- | --- |
|  |  | *pH 6.0* | *pH 6.5* | *pH 7.0* | *pH 6.0* | *pH 6.5* | *pH 7.0* | |
| CFU aerob  [10^8^ CFU mL^-1^] | 0.02 ± 0.01 | 0.9 ± 0.2 | 3 ± 0.3 | 1 ± 1 | 3 ± 3 | 3 ± 2 | | 50 ± 20 |
| CFU anaerob [10^9^ CFU mL^-1^] | 0.1 ± 0.03 | 8 ± 2 | 12 ± 7 | 6 ± 2 | 8 ± 2 | 10 ± 7 | | 6 ± 2 |
| Σ SCFA  [mg mL^-1^] | 5.12 ± 0.14 | 8.52 ± 0.48 | 8.68 ± 0.24 | 8.90 ± 0.36 | 7.92 ± 0.40 | 8.43 ± 0.39 | | 8.14 ± 0.36 |
| Acetate  [mg mL^-1^] | 2.74 ± 0.07 | 3.14 ± 0.17 | 3.82 ± 0.14 | 4.07 ± 0.22 | 4.37 ± 0.30 | 4.35 ± 0.26 | | 4.16 ± 0.59 |
| Butyrate  [mg mL^-1^] | 0.99 ± 0.03 | 1.90 ± 0.09 | 1.57 ± 0.16 | 1.38 ± 0.17 | 0.94 ± 0.06 | 1.42 ± 0.13 | | 0.87 ± 0.07 |
| Propionate  [mg mL^-1^] | 1.15 ± 0.04 | 3.03 ± 0.20 | 2.94 ± 0.13 | 3.05 ± 0.21 | 2.39 ± 0.52 | 2.51 ± 0.19 | | 2.96 ± 0.43 |
| Iso-valerate  [mg mL^-1^] | 0.24 ± 0.00 | 0.45 ± 0.13 | 0.36 ± 0.08 | 0.40 ± 0.18 | 0.21 ± 0.06 | 0.16 ± 0.06 | | 0.15 ± 0.03 |
| Richness  [-] | 99 | 100 ± 6 | 99 ± 7 | 126 ± 36 | 75 ± 23 | 104 ± 11 | | 81 ± 14 |
| Shannon effective  [-] | 29 | 22 ± 4 | 24 ± 1 | 24 ± 5 | 25 ± 8 | 24 ± 6 | | 18 ± 6 |
| Actinobacteria  [%] | 0.65 | 0.33 ± 0.09 | 0.68 ± 0.09 | 0.17 ± 0.10 | 1.18 ± 0.92 | 0.04 ± 0.03 | | 0.02 ± 0.02 |
| Bacteroidetes  [%] | 48.79 | 58.81 ± 8.32 | 65.88 ± 6.82 | 71.90 ± 3.62 | 57.32 ± 3.70 | 75.01 ± 0.79 | | 70.35 ± 5.79 |
| Firmicutes  [%] | 45.28 | 36.74 ± 10.39 | 27.40 ± 8.32 | 22.92 ± 5.58 | 37.23 ± 1.30 | 22.27 ± 0.23 | | 23.61 ± 6.16 |
| Proteobacteria  [%] | 1.59 | 3.50 ± 1.54 | 5.14 ± 1.73 | 8.65 ± 3.12 | 3.27 ± 2.95 | 3.96 ± 2.02 | | 4.05 ± 1.84 |
| Verrucomicrobia  [%] | 2.30 | 0.59 ± 0.97 | 0.34 ± 0.38 | 0.48 ± 0.40 | < 0.01 | < 0.01 | | < 0.01 |
| *Akkermansia*  [%] | 1.98 | 0.03 ± 0.03 | 0.34 ± 0.38 | 0.48 ± 0.40 | < 0.01 | < 0.01 | | < 0.01 |
| *Bacteroides*  [%] | 38.51 | 54.33 ± 5.50 | 64.83 ± 6.82 | 70.00 ± 3.40 | 48.70 ± 14.23 | 63.83 ± 15.87 | | 70.79 ± 9.97 |
| *Bifidobacteria*  [%] | 0.35 | 0.27 ± 0.07 | 0.62 ± 0.80 | 0.17 ± 0.09 | 1.16 ± 0.93 | 0.03 ± 0.02 | | 0.02 ± 0.02 |
| *Blautia*  [%] | 1.05 | 0.59 ± 0.05 | 0.36 ± 0.22 | 0.11 ± 0.07 | 0.97 ± 0.60 | 1.00 ± 0.73 | | 0.26 ± 0.27 |
| *Faecalibacterium*  [%] | 6.39 | 10.04 ± 1.01 | 0.56 ± 0.36 | 0.44 ± 0.43 | 0.01 ± 0.005 | 1.03 ± 0.35 | | 0.01 ± 0.01 |
| *Roseburia*  [%] | 4.18 | 0.02 ± 0.01 | 0.01 ± 0.02 | 0.01±0.01 | < 0.01 | < 0.01 | | < 0.01 |
| *Clostridium*  Cluster XIVa [%] | 0.19 | 4.70 ± 0.60 | 3.94 ± 1.09 | 2.74 ± 1.79 | 2.62 ± 1.80 | 5.05 ± 1.21 | | 4.21 ± 1.78 |
| *Escherichia/ Shigella* [%] | 0.24 | 2.43 ± 0.16 | 4.94 ± 1.94 | 0.52 ± 0.16 | 2.40 ± 2.36 | 2.57 ± 1.42 | | 3.24 ± 2.47 |
